# Fracture Characterization of Ultra-High Performance Concrete Notched Beams under the Influence of Different Material Factors Based on Acoustic Emission Technique

**DOI:** 10.3390/ma14164608

**Published:** 2021-08-17

**Authors:** Xianqiang Wang, Duo Liu, Yao Zhang, Yubo Jiao

**Affiliations:** 1 The State Key Laboratory on Safety and Health of In-Service Long-Span Bridges, Institute of Transportation Science, JSTI Group, Nanjing 211112, China; wxq350@jsti.com (X.W.); liuduojsti@163.com (D.L.); 2 School of Civil Engineering, Southeast University, Nanjing 211189, China; 3 Key Laboratory of Urban Security and Disaster Engineering of Ministry of Education, Faculty of Architecture, Civil and Transportation Engineering, Beijing University of Technology, Beijing 100124, China; zhangyaobjut@163.com

**Keywords:** UHPC, notched beam, fracture characterization, AE, material factor influence

## Abstract

Acoustic emission (AE) technology is widely used in structural health monitoring. Glass sand (GS) made from waste glass is a promising replacement aggregate for quartz sand (QS) in ultra-high performance concrete (UHPC). This paper addresses the effects of different factors including water-binder ratio, length of basalt fiber (BF) and ratio of GS replacing QS on the fluidity and flexural strength of UHPC notched beam under four-point flexural loads. Meanwhile, the fracture characteristics of UHPC notched beam were characterized through acoustic emission (AE) technique. The results show that water-binder ratio and replacement ratio of GS present a positive correlation with work performance of UHPC, while length of BF exhibits a negative one. The flexural strength of UHPC notched beams can be improved by the decrease of the water-binder ratio and fiber length. The effect of water-binder ratio on flexural strength is the most significant, while the addition of GS presents the minimum one. The fracture characteristics of UHPC notched beams could be favorably characterized by AE parameters. Through the analysis and comparison of the evolution of AE parameters, the differences in fracture properties of UHPC notched beams with different flexural strengths can be realized. Through this study, the fluidity and flexural performance of UHPC produced by replacing QS with GS were demonstrated, which is beneficial to the cleaner production of UHPC. Meanwhile, the AE technique presented great potential for fracture characterization of UHPC notched beam, which also provided a promising method for real-time monitoring of cracking in the diagnosis of UHPC structures.

## 1. Introduction

Ultra-high performance concrete (UHPC) is an advanced cement-based composite material with ultra-high mechanical properties and excellent durability [1,2,3]. It has been successfully used in bridges, high-rise buildings, nuclear power plants, gymnasiums and other large buildings [4,5,6]. The properties of raw materials play a key role in the mechanical properties of UHPC. The setting of low water-binder ratio and fine aggregate size make UHPC exhibit excellent compactness. The use of fibers with high tensile properties makes UHPC possess ultra-high tensile and flexural strength. The ultra-high mechanical properties of UHPC have been widely studied by changing the properties of various raw materials. Along with the decrease of water-binder ratio, the tensile strength (including matrix crack strength and composite failure strength) and compressive strength were enhanced when the sand-binder ratio was at an appropriate level (0.3–0.8) according to the study from Ye et al. [7]. Shin et al. [8] optimized the mix proportion and composition of 180 MPa UHPC. The results showed that the mechanical properties were improved by the decrease of water-binder ratio when the working performance was guaranteed. The mechanical properties of UHPC with different steel fiber lengths (8, 12 and 16 mm) were studied by Abbas et al. [9]. It showed that the super-high PC mixture with short steel fiber exhibited better flexural resistance compared with the long steel fiber with similar volume. In order to evaluate the flexural and thrust resistance of the concrete lining section of the tunnel, the flexural and edge-point load tests on a one-third-scale fiber-reinforced concrete tunnel lining section were carried out [10]. The results showed that the short fiber (8 mm) tunnel lining segment has higher cracking and peak load compared with the long fiber (16 mm) tunnel lining segment. The effect of expensive silica sand completely replaced by recycled glass cullet or two local natural sands was demonstrated by Yang et al. [11]. The results showed that the fluidity of fresh ultra-high performance fiber-reinforced concrete (UHPFRC) can be reduced by using more angular sand, and local natural sand can produce mechanical properties and ductility of UHPFRC similar to silica sand.

The conventional aggregate used in UHPC is quartz sand (QS). The production of QS is based on crushing coarse sand or rock, which is time-consuming, costly and polluting by dust during crushing. Therefore, new aggregate which meets the strength requirements and environmental protection has become a green development trend in UHPC. Waste glass accounts for a large proportion of municipal solid waste (MSW), whose disposal in landfill causes serious environmental pollution. Recycling and utilization of waste glass are beneficial to save primary raw materials and reduce CO_2_ emissions. Glass sand made from waste glass presents many advantages including cost-effective, chemical stability, low absorptivity and similar density to natural gravel, which is regarded as a promising replacement material in cement-based material [12]. The feasibility of replacing quartz sand (QS) with glass sand (GS) in UHPC was studied by Soliman and Tagnit-Hamou [13]. In their study, an optimal grading curve was recommended. Experimental results revealed that both partial (50% in weight) and total replacements of QS with GS enhanced the compressive strength of UHPC. Jiao et al. [12] demonstrated the influence of replacing quartz sand with glass sand on working performances and mechanical properties. The results reveal that the addition of glass sand is beneficial to the fluidities and compressive strength of UHPC. However, the research on the mechanical properties of UHPC notched beam with different material variables and levels is limited. The influence of various material variables on the flexural strength of UHPC notched beams and the influence degree of these variables need to be further studied.

The commonly used methods to characterize the internal damage mechanism of UHPC are X-ray [14], ultrasonic [15,16,17], acoustic emission (AE) [18,19,20], etc. AE technology has been widely used in the detection of concrete beam fracture process, because the fracture evolution of structure can be reflected by AE parameters without additional assumptions. The experimental results showed that the damage behavior of UHPC specimen can be well described by the calibration Weibull function based on AE signal [21]. From parametric studies between rise angle and average frequency [22], it is found that AE analysis can be successfully used to determine crack movement and classify damage level of retrofitted beams. Investigation on fracture process zones in notched concrete beams under three-point bending was performed by applying AE [23]. The widths of the fracture process zone were estimated from results of AE source location. It is confirmed that the fracture energy correlated with the width of AE cluster, as the energy increased when the width of fracture process zone expanded. The degradation mechanism of concrete beams under bending load was studied by using AE technology [24]. Empirical laws describing the relationship between damage evolution and AE activities were proposed in order to predict the degradation state of the material from AE activities. The crack growth process of precast concrete beam under three-point bending condition was monitored by using AE technology and resonance frequency extraction technology [25]. The results showed that the mode of crack growth can be analyzed by average frequency and rise angle value, and the process of crack growth and damage can be monitored by cumulative AE energy and variation in the resonant bending frequencies. In order to study the influence of loading rate on the fracture behavior of concrete, three-point bending fracture tests were carried out on concrete with different loading rates, and AE technology was used for real-time monitoring [26]. The results showed that the width of concrete crack can be represented by the number of AE events, the change of fracture energy under different loading rates can be represented by AE cumulative energy, and the failure forms under different loading rates can be analyzed by the change of *b*-value. In the existing research, the AE parameters exhibit a good interpretation and characterization effect on the fracture phenomenon and the change of fracture parameters, but the characterization on the whole process of internal structure damage and fracture is limited. Moreover, the existing research relatively lacks the characterization and comparison on the fracture process of UHPC notched beams with different flexural strengths.

In this paper, the effects of water-binder ratio (0.17, 0.19 and 0.21), length of basalt fiber (BF) (6, 13 and 20 mm) and the replacement ratio of GS (0, 50 and 100%) to quartz sand on the working performance and flexural strength of UHPC notched beams were studied. The fracture process of UHPC notched beams was characterized and compared by AE analysis. UHPC notched beams with different combinations of factors’ levels were prepared. The slump test was used to evaluate the working performance. The four-point bending test was used to evaluate the flexural performance. Meanwhile, the fracture characteristics of UHPC notched beams were measured synchronously by AE technology, and the corresponding fracture properties were characterized and compared by AE parameters including impact number, amplitude, energy and *b*-value.

## 2. Materials and Methods

### 2.1. Raw Materials

In this study, P.O 52.5 cement manufactured by Yangchun Cement Ltd, Zhucheng, China conforming to GB 175–2007 [27] was used to produce all concrete mixtures. The adopted silica fume (SF) met the requirements of GB/T 27690-2011 [28]. QS and GS aggregates were screened to maintain the same continuous particle size distribution (PSD). The average particle size of the two sands was set to 300 μm. Meanwhile, the density of UHPC could be improved by using quartz powder (QP) with an average particle size of 15 μm. The chemical and physical properties of cement, SF, QS, GS and QP, the PSD curves of cement, SF, QS, GS and QP and particle mixture in UHPC can be found in the study by Jiao et al. [12]. All UHPC specimens had the same PSD curve, because QS and GS with the same particle size distribution were used in this study. BF with the lengths of 6, 13 and 20 mm were added to the mixtures. A based polycarboxylic high performance water reducer admixture (HPWRA), with specific gravity of 1.1 (20 °C) and solid content of 20%, was added to UHPC mixture. The appearances of QS, GS and BF can also be found in the study by Jiao et al. [12].

### 2.2. Proportion of Mixes

The response surface method (RSM) has been widely used in experimental design and variable analysis of engineering materials [29,30,31]. The variables are a combination of three factors with three levels respectively in this study. The three-level Box-Behnken design with three factors (water-binder ratio; length of BF; replacement ratio of GS) was carried out. The software Design-Expert 8.0 was adopted for the scheme design, model generating, statistical analysis and optimization of the preparation parameters. According to the software design result, 17 groups of test arrangements were obtained, and the mix proportion of raw material with each group of tests is listed in Table 1.

### 2.3. Specimen Preparation

To achieve a homogeneous structure and avoid particle agglomeration, all concrete mixtures were mixed by the mixer according to the following procedures: (1) Dry components, including cement, SF, aggregate and QP, were poured into the mixer according to the proportion of the mixture listed in Table 1, stirred at low speed for 3 min before the addition of water and HRWRA; (2) half of the HRWRA diluted in half of the water was gradually added over 2 min of mixing time. The remaining water and HRWRA were gradually added during the other 2 min of mixing; (3) BF was slowly added and mixed for another 8 min until achieving a uniform distribution.

At the end of mixing, the fresh properties of concrete mixtures were measured using slump test to characterize their workability. Meanwhile, fresh mixtures were cast into molds and kept at 20 °C and 95% RH for 24 h. They were then demolded and cured in a standard curing room with temperature of 20 °C and 95% RH until 28 days. Three replicated samples were prepared for each test and the corresponding average value was treated as the representative one.

### 2.4. Specimen Preparation

#### 2.4.1. Slump Test

Slump measurement of fresh mixture was used to evaluate the fluidity and workability of all concrete mixes. The operation procedure was conducted in accordance with the slump test standards [32,33].

#### 2.4.2. Four-Point Bending Test

The four-point bending test was carried out according to relevant test standards [32,34]. Concrete prisms with dimensions of 100 × 100 × 400 mm were used for tests. The 30T Mechanical Testing & Simulation (MTS) machine was used to test the flexural strength under the equal displacement loading speed setting of 0.2 mm/min. The middle of the bottom span of the test piece was a notch with a width of 2 mm and a depth of 20 mm. The notch was obtained by wedging a steel piece into the mold for one hour and then pulling the steel piece out. The flexural strength was determined by Formula (1).

(1) $$f_{f}=\frac{Fl}{bh^{2}}$$

where $f_{f}$ is the flexural strength of concrete, MPa; F is the failure load of specimens, N; l is the span between supports, mm; h is the effective height of specimens, equal to the height of the test piece minus the depth of the notch, mm; b is the width of specimens, mm.

#### 2.4.3. AE Test

The phenomenon of transient elastic waves generated by the rapid release of energy from local sources in materials is called the AE phenomenon. The AE wave propagates through the material to the sensor installed on the structure surface. The AE wave can be converted into electrical signal by the sensor. The damage state of the structure can be detected by amplifying the electrical signal for data analysis.

**Figure 1:**
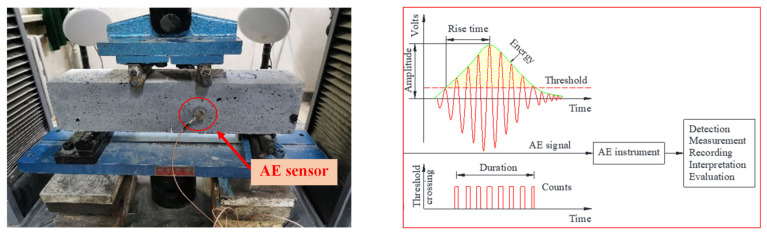
AE tests of UHPC notched beams under four-point bending and corresponding AE parameters.

In this study, a twelve-channel SAEU2S data acquisition system from the Soundwel Technology Corporation was used. A sensor with a frequency range of 22–220 kHz and resonant frequency of 150 kHz was firmly attached to the side of each sample by a coupling agent, and the AE signal was collected during the four-point bending test. The threshold value of the AE acquisition system was set to 45 dB, and the acquisition frequency was set to 5 msps. The AE parameters including impact number, amplitude, ringing count, rise time, duration and energy were collected. AE testing arrangement for UHPC notched beams under four-point bending is illustrated in Figure 1.

In this study, the AE parameters (amplitude, energy) which can be directly collected and the cumulative AE parameters (cumulative amplitude and energy) were used to characterize the fracture characteristics of UHPC notched beams under four-point bending. Signal amplitude is the magnitude of peak voltage of the largest excursion attained by the signal waveform from a single AE event [35], which is the indicator of signal size. Signal energy is defined as the measured area of the rectified AE signal [35], which is associated to intensity of damage. It can be calculated by

(2) $$E=\int_{0}^{t}V^{2}(t)dt$$

where V(t) is the recorded voltage, t is duration time.

The AE signal that exceeds the threshold and makes a channel obtain data was called an impact, impact number was often used to characterize the density of cracks in beams.

Based on the evaluation indexes of impact number and energy, the value of $ID^{H}$ represent the ratio of the fractional impact number with the single load level to the cumulative impact number with the whole test process. Similarly, the value of $ID^{E}$ represents the ratio of the fractional energy with the single load level to the cumulative energy with the whole test process. These two parameters were used to analyze and compare the fracture characteristics of concrete notched beams with different flexural strengths.

The existing research showed that there was a certain relationship between cracks type and *b*-value in the fracture process of concrete specimens. Generally, in the process of micro cracks forming, the *b*-value increases, but in the subsequent combination of these micro and large cracks, the *b*-value decreases gradually, and the *b*-value is calculated by

(3) $$\log_{10}N=a-b\left(\frac{A_{dB}}{20}\right)$$

where $A_{dB}$ is the peak amplitude of AE event expressed in decibels, *N* is the incremental frequency, *a* and *b* are the empirical constants obtained by linear fitting.

## 3. Results and Discussions

### 3.1. Slump and Flexural Strength Analysis Based on RSM

#### 3.1.1. Experimental Scheme

The three-level Box-Behnken design with three factors (water-binder ratio, X1; length of BF, X2; replacement ratio of GS, X3) was carried out. Actual and coded values of the independent variables are shown in Table 2. Combinations of X1 (0.17, 0.19, 0.21), X2 (6, 13, 20 mm) and X3 (0%, 50%, 100%) were selected as independent variables. Slump, Y1; flexural strength, Y2, were adopted as the responses. The scheme consisted of 17 experimental groups, including five repeats of central point, which was less than a complete experimental plan 3^3^ = 27. Three test pieces are used in a group, and their corresponding average value is taken as the representative value. The experimental results are shown in Table 3. The relationship between the independent variable and the response value is described by the 3D response surface graph. A 3D plot is used to represent the dependent variables in function of two independent parameters, when other variables are kept constant at the central point.

As shown in Table 3, the values of slump ranged from 44 to 112 mm, and the values of flexural strength ranged from 11.39 to 15.42 MPa. The minimum and maximum values of slump were obtained from group 10 (*X*_1_: 0.17; *X*_2_: 13 mm; *X*_3_: 0%) and group 9 (*X*_1_: 0.21; *X*_2_: 6 mm; *X*_3_: 50%), group 15 (*X*_1_: 0.19; *X*_2_: 6 mm; *X*_3_: 100%) respectively, and the minimum and maximum values of flexural strength were obtained from group 6 (*X*_1_: 0.21; *X*_2_: 20 mm; *X*_3_: 50%) and group 14 (*X*_1_: 0.17; *X*_2_: 6 mm; *X*_3_: 50%) respectively.

#### 3.1.2. Analysis of Variance (ANOVA) Results for Models and Independent Variables

The significance of the models constructed for slump and flexural strength was checked by coefficient of determination (*R*^2^), adjusted coefficient of determination (Adj. *R*^2^), Adeq. precision, and Fisher’s test value (*F* value). Models and factors were considered significant when *p* < 0.05. ANOVA results for models and independent variables are obtained and listed in Table 4 and Table 5.

#### 3.1.3. Slump

As shown in Table 4, the result of ANOVA for slump showed that the model possessed a satisfactory level of *R*^2^ (0.7992), Adj. *R*^2^ (0.7529), and Adeq. precision (12.434), which were significant at *p* < 0.0001. The *R*^2^ of 0.7992 was in reasonable agreement with the Adj. *R*^2^ of 0.7529. Adeq. Precision measures the signal-to-noise ratio, a ratio greater than 4 is desirable. The ratio of 12.434 with this model indicated an adequate signal. This model can be used to navigate the design space. 

As can be seen from the Table 5, the significant variables (*p* < 0.05) for the penetration model included *X*_1_, *X*_2_, *X*_3_. A linear slump model based on coding factor was obtained by removing unimportant variables:

(4) $$Y_{1}=78.82+14.13{\;\times \;X}_{1}\;-\;12.75{\;\times \;X}_{2}+13.63{\;\times \;X}_{3}$$

In the slump model, the most significant variable was the water-binder ratio, the second was the replacement ratio of GS, and the last was the length of BF. The three variables were all significant with *p*-values lower than 0.05.

**Figure 2:**
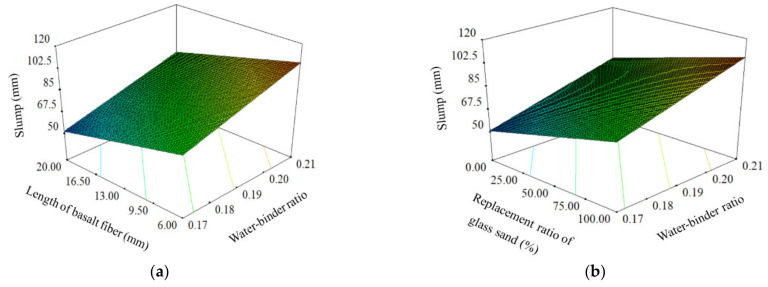
3D response surface plots between preparation parameters and slump: (**a**) Length of basalt fiber and water-binder ratio; (**b**) replacement ratio of glass sand and water-binder ratio.

The relationships between preparation parameters and slump were demonstrated in 3D response surface plots, which are shown in Figure 2. As shown in the figure, there was a positive correlation between water-binder ratio, replacement ratio of GS and slump. The increase of water-binder ratio or replacement ratio of GS will lead to the increase of slump, which meant that the working performance of UHPC will be enhanced. It is obvious that the increase of water-binder ratio will enhance the working performance. The working performance can be improved by increasing the replacement ratio of GS, because the GS has lower water absorption than QS. There was a negative correlation between length of BF and slump. It showed that the slump will be reduced when the length of BF increased, and the working performance of UHPC will be reduced. When fiber length increased, the fluidity of concrete was restricted because the area of cement paste connected with the fiber enlarged, which will cause a decrease in slump value.

#### 3.1.4. Flexural Strength

As shown in Table 4, the result of ANOVA for flexural strength showed that the model possessed a satisfactory level of *R*^2^ (0.8377), Adj. *R*^2^ (0.6289) and Adeq. precision (5.895), which were significant at *p* < 0.05. The *R*^2^ of 0.8377 was in reasonable agreement with the Adj. *R*^2^ of 0.6289. Adeq. Precision measures the signal-to-noise ratio, a ratio greater than 4 is desirable. The ratio of 5.895 with this model indicated an adequate signal. This model can be used to navigate the design space. 

As can be seen from the Table 5, the significant variables (*p* < 0.05) for the penetration model included *X*_1_, *X*_2_, *X*_11_. A second-order flexural strength model based on coding factor was obtained by removing unimportant variables:

(5) $$Y_{2}=12.20\;-\;0.90{\;\times \;X}_{1}\;-\;0.66{\;\times \;X}_{2}+1.50{\;\times \;X}_{11}$$

In the flexural strength model, the most significant variable was the water-binder ratio, the second was the length of BF. The two variables were all significant with *p*-values lower than 0.05. The effect of replacement ratio of GS was not significant.

**Figure 3:**
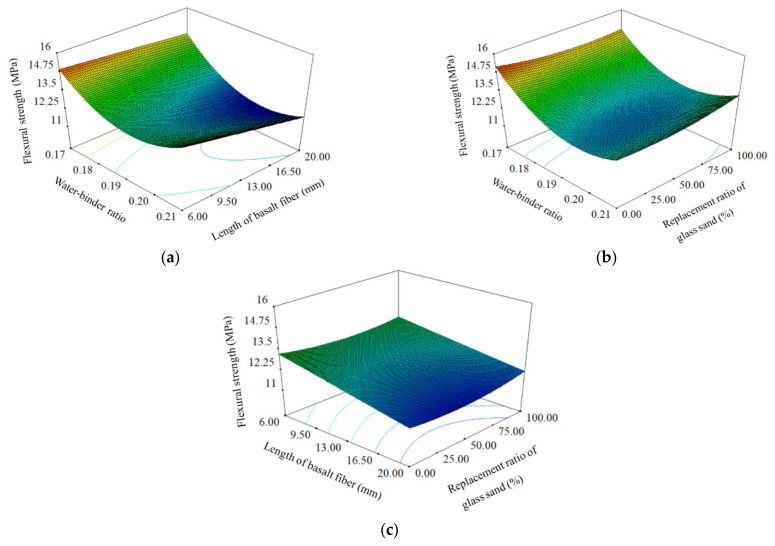
3D response surface plots between preparation parameters and flexural strength: (**a**) Water-binder ratio and length of basalt fiber; (**b**) water-binder ratio and replacement ratio of glass sand; (**c**) length of basalt fiber and replacement ratio of glass sand.

The relationships between preparation parameters and flexural strength were demonstrated in 3D response surface plots, which are shown in Figure 3. Water-binder ratio and length of BF were proved to have negative correlation with flexural strength. The flexural strength of UHPC notched beam can be improved by the decrease of water-binder ratio or length of BF. The internal structure of the notched beam will be more compact when the water-binder ratio decreased, which ensured the specimen has a stronger ability to resist bending force. The decrease of fiber length meant the increase of fiber number under the same content. The BF plays a key role in resisting the bending force, and the flexural strength was improved because a large number of short fibers form a stronger force to resist the load. The influence of GS on the flexural strength of notched beam was not significant because the ultimate failure load of specimen subjected to four-point bending mainly depended on the compactness of structure and reinforcement effect of BF.

### 3.2. Fracture Analysis Based on AE Parameters

#### 3.2.1. Fracture Characteristics Based on Amplitude and Energy

The fracture characteristics of UHPC notched beams with different strengths were characterized and compared by using AE parameters. These AE parameters include amplitude, energy, cumulative amplitude and cumulative energy. The representative specimens were selected according to the change rule of flexural strengths. Three notched beam specimens with flexural strength ranging from 15.42 to 11.39 MPa were selected in this study.

**Figure 4:**
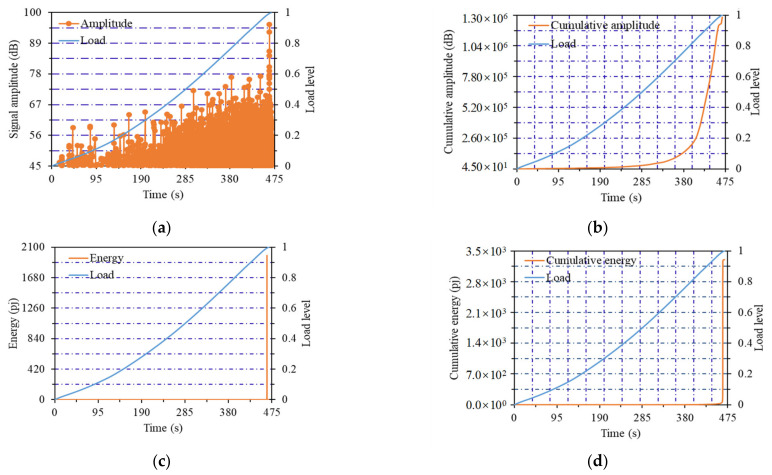
Curves of acoustic emission parameters of No.14 notched beam: (**a**) Amplitude curve; (**b**) cumulative amplitude curve; (**c**) energy curve; (**d**) cumulative energy curve.

**Figure 5:**
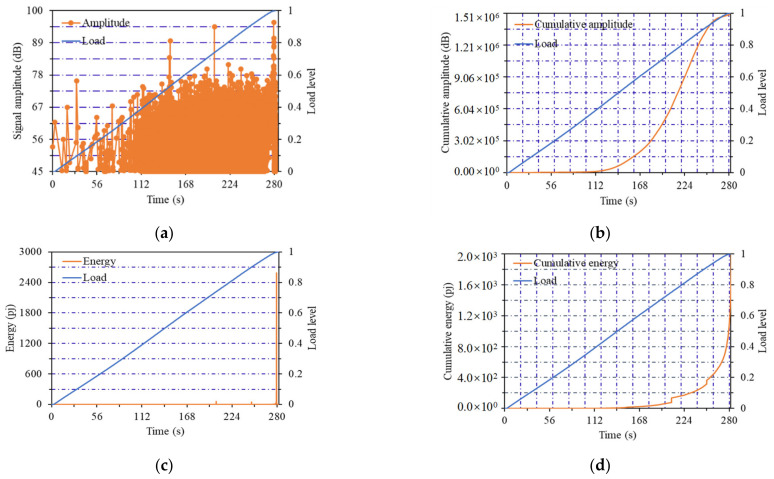
Curves of acoustic emission parameters of No.11 notched beam: (**a**) Amplitude curve; (**b**) cumulative amplitude curve; (**c**) energy curve; (**d**) cumulative energy curve.

**Figure 6:**
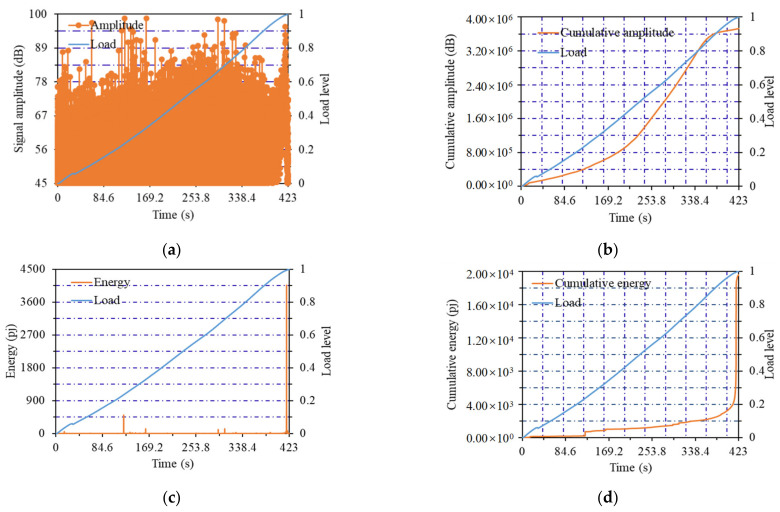
Curves of acoustic emission parameters of No. 6 notched beam: (**a**) Amplitude curve; (**b**) cumulative amplitude curve; (**c**) energy curve; (**d**) cumulative energy curve.

The change curves of AE parameters of the No.14 notched beam specimen (listed in Table 1) with flexural strength of 15.42 MPa are shown in Figure 4. The change curves of AE parameters of the No.11 notched beam specimen with flexural strength of 12.83 MPa are shown in Figure 5. The change curves of AE parameters of the No.6 notched beam specimen with flexural strength of 11.39 MPa are shown in Figure 6. Notched beams with high, medium and low flexural strength were represented by these three specimens, respectively.

As shown in Figure 4a, when the flexural strength of the notched beam specimen was the high value of 15.42 MPa, the first stage of the amplitude curve was the load from 0 to 0.9 load level, and the amplitude showed a steady upward trend. It should be noted that the upward trend of the amplitude and load curves almost kept the same. This phenomenon can be interpreted as the internal structure of beams with higher flexural strength was relatively dense, and the probability of great fluctuation of crack development was small. The development of internal structure crack was consistent with the increase of load, which explained the development trend of amplitude value in this stage. When load reached the 0.9 level, the amplitude curve entered the second stage (i.e., the sudden change stage), and the amplitude value suddenly increased until load reached the maximum value. When the load was close to the failure load, the internal cracks developed into macro cracks, which led to the sudden increase of amplitude value.

In Figure 4b, it is presented that the first stage of the cumulative amplitude curve was from the beginning of loading to the 0.5 load level, the curve was very gentle and the amplitude growth was not obvious. At this stage, the dense internal structure was very effective to resist the load. The increase of the curve slope started from the 0.5 load level, at this time, it entered the second stage, the number of amplitude values started to increase at a slower speed. It can be seen that the internal fractures began to exhibit obvious generation and development. When the load reached the 0.8 level, the cumulative amplitude curve entered the third stage and the slope of the curve increased obviously, which meant that the amplitude entered the high growth stage until the failure load reached. A large number of internal cracks began to develop and penetrate, resulting in the rapid emergence of amplitude value. 

From the changes of energy curve and cumulative energy curve in Figure 4c,d, it can be observed that the obvious generation and mutation of energy were at the last 0.9~1 load level. Certain brittleness will be reflected in the final failure of the notched beam due to the existence of the notch. This phenomenon will be more obvious when the strength of the beam was greater, which led to the sudden increase of energy under the final load level. The energy generated by the cracks under the previous load levels was covered by the final extremely high energy value.

As shown in Figure 5a, when the flexural strength of the notched beam specimen was the middle value of 12.83 MPa, the first stage of amplitude curve was from 0 to 0.4 load level, a small amount of high amplitude values generated at this stage. This showed that the internal structure of the lower strength notched beam was not dense enough, and a small amount of AE signal was collected when the original clearances were squeezed. When the load increased to 0.4 load level, the curve came to the second stage. At this stage, the amplitude curve maintained at a high level of about 73 dB until the load reached 0.9 level, whereby the cracks entered a stable development stage. The third stage entered when the load reached 0.9 load level, where the amplitude suddenly changed to more than 90 dB until the test piece was damaged. When the load was close to the failure load, the higher signals the internal crack occurred were collected, which led to the sudden increase of amplitude value.

From Figure 5b, it can be seen that the first stage of the cumulative amplitude curve was from the beginning of loading to the 0.4 load level, the curve was very gentle and the increase of amplitude quantity was not obvious. The AE signal from the original clearances was very small, which can correspond to the amplitude curve. This trend was similar to that of high-strength notched beam at this stage. The increase of the curve slope started at 0.4 load level, which meant that the curve entered the second stage and the amount of amplitude started to increase substantially. This was the result of a large number of high amplitude values produced by the stable development of cracks. When the load reached 0.7 level, the curve entered the third stage, the slope of curve increased obviously and the amplitude entered the high growth stage until reached 0.9 load level; the development of cracks was more severe at this stage. It can be found that the load levels of the second and third stages were earlier than that of the high-strength notched beam, which can be explained as the decrease of the strength led to the development of earlier and more severe cracks. When the load reached the 0.9 load level (i.e., the fourth stage), the slope of the curve decreased gradually and the amplitude increased slowly until the maximum load level. A large number of cracks developed at the front load level when the strength of the specimen was not high enough, and the amplitude value at the last stage tended to slow down rather than continue to maintain rapid growth.

From the energy curve in Figure 5c, it can be observed that the sudden change of energy was also located at the failure time of 0.9–1.0 load level, but the difference was that a very small amount of energy values occurred at the previous 0.7–0.9 load level compared with the high-strength notched beam. Low energy values were collected because the cracks developed at the early load level. 

The cumulative energy value curve is shown in Figure 5d. It showed that the first stage was the load from 0 to 0.6 level, the curve was gentle and no obvious energy value was generated at this stage. In the second stage, the load reached 0.6 load level, the curve slope began to increase with a continued increasing trend, and the energy value started to generate and gradually entered into the stage of rapid growth until the maximum load was reached. This showed that, although the high energy value covered the low energy value generated at the previous load level in Figure 5c, the generation process of the energy value also had a trend with increasing speed. This phenomenon also indicated that the cracks developed more fully at the earlier load level when the notch beam strength decreased.

As shown in Figure 6a, when the flexural strength of the notched beam specimen was the low value of 11.39 MPa, the amplitude curve could be roughly divided into two stages. The first stage was from the beginning of loading to the 0.9 load level, and the overall trend of the amplitude curve presented a “wave shape” that maintained a high signal value of 75 db, the high signal value generated frequently during the loading process. The notched beam with low flexural strength had more internal clearances and the notch made more use of the development of these cracks, which made the amplitude value maintain at a high level with wave development. The second stage was from 0.9 load level to the maximum load, and the amplitude value suddenly increased to more than 90 dB until the maximum load at this stage, and this phenomenon was the same as that of high-strength and medium-strength notched beams.

The cumulative amplitude curve is shown in Figure 6b. It showed that the first stage was from the beginning of loading to the 0.4 load level, and the slope of the curve began to increase, that was, the increase of amplitude occurred since the beginning of loading. This was due to the pressure on a large number of initial clearances. When the loading reached 0.4 load level, the slope of the curve increased significantly, and the amplitude entered the second stage of high growth, the cracks began to enter the rapid development stage and the cracks continued to expand. When loading entered the 0.9 load level, the curve entered the third stage. At this stage, the slope of the cumulative amplitude decreased gradually, which meant the amplitude value generation was relative slow until the maximum load level and the specimen was damaged. It was the same trend as the medium strength notched beam at this stage.

From the curve of energy value in Figure 6c, it is shown that the sudden increase of energy was also located at the last failure time, but a higher energy value of 450 pj was generated in the earlier 0.2 load level, and a certain amount of energy values were generated in the process from 0.2 load level to the time of sudden increase. The generation of large cracks occurred at a more forward loading level, which resulted in the earlier generation of high energy values when the flexural strength was the lowest value.

The cumulative energy curve is shown in Figure 6d. It can be seen that the first stage was the 0~0.2 load level, the curve was gentle and there was no obvious energy value generated. When loading reached 0.2 load level, the curve came to the second stage, the curve slope began to increase and kept the trend until 0.95 load level. Corresponding to the energy curve, high energy values were generated at an early stage, which made the slope of the cumulative energy value increase. It can be seen that the load level corresponding to energy generation was more advanced than that of the beam with medium strength notch. In the third stage, the load reached 0.95 load level, the energy value suddenly increased, and the curve rose sharply and straightly until the maximum load was reached, whereby the specimen was damaged. This is the stage that the internal cracks transformed into a large number of macro cracks, and a large number of energy values were collected.

#### 3.2.2. Fracture Characteristics Based on ${ID}^{H}$ and ${ID}^{E}$

**Figure 7:**
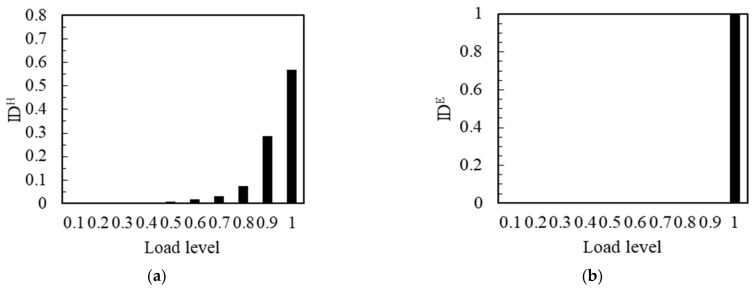
$ID^{H}$ and $ID^{E}$ of the No. 14 notched beam: (**a**) $ID^{H}$; (**b**)$\;ID^{E}$.

The representative specimens were selected according to the change rule of flexural strength. As shown in Figure 7a, when the flexural strength of the notched beam specimen was the high value of 15.42 MPa, the $ID^{H}$ was very small, from 0.1 to 0.4 load level, and it could hardly be observed. It meant that the relative number of impacts was very few at this stage. The strong load resistance was reflected in the high strength notched beam, which resulted in less impact number at the low load levels. When the load level reached 0.5, the value of $ID^{H}$ began to reflect, and kept rising until the maximum load. At this stage, the number of impacts started to increase and continued this trend until the moment of maximum load. The maximum impact number occurred in the final 1.0 load level. This phenomenon can correspond to the synchronous effect of cracks development and load increase when the strength of the notched beam was high enough. As shown in Figure 7b, the $ID^{E}$ was almost only reflected and mostly filled in the 1.0 load level. It showed that the energy was almost only reflected in the final load level in the experimental process. This also showed the brittleness of the high strength notched beam.

**Figure 8:**
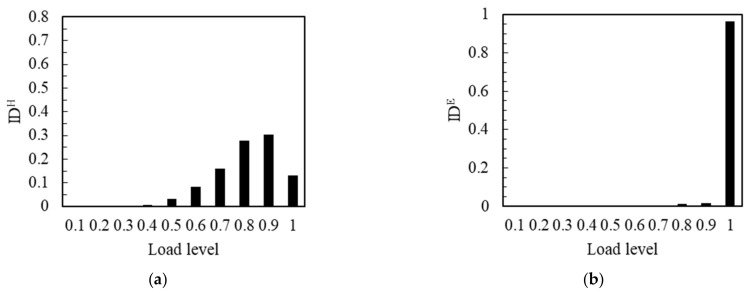
$ID^{H}$ and $ID^{E}$ of the No.11 notched beam: (**a**) $ID^{H}$; (**b**)$\;ID^{E}$.

The $ID^{H}$ corresponding to the flexural strength of the notched beam specimen with the middle value of 12.83 MPa is shown in Figure 8a. From 0.1 to 0.3 load level, the value of $ID^{H}$ was very small and almost invisible, which meant that the relative impact number was small at this stage. $ID^{H}$ increased from 0.4 load level, the impact number started to increase continuously from 0.4 to 0.9 load level at this stage. $ID^{H}$ reached the maximum at 0.9 load level. It was worth noting that the load that can be effectively resisted was lower and the apparent time of the number of impacts was earlier when the strength of the notched beam decreased. When the load exceeded 0.9 level, the value of $ID^{H}$ decreased rapidly in the last load level. It meant that the impact number decreased significantly in 1.0 load level. This can correspond to the previous cumulative amplitude curve, the slowing of the cumulative curve meant the decrease of the effective signal number, which in fact means the decrease of the impact number. That was to say, the development of a large number of cracks has been carried out before the final load level. As shown in Figure 8b, the very small value of $ID^{E}$ occurred at 0.8 and 0.9 load levels, and most of the energy was generated at the 1.0 load level. This was essentially the same as the energy curve and the cumulative energy curve. This showed that, although the number of cracks that developed in the final load was relatively small, the extremely high energy was released during the penetration of these cracks.

**Figure 9:**
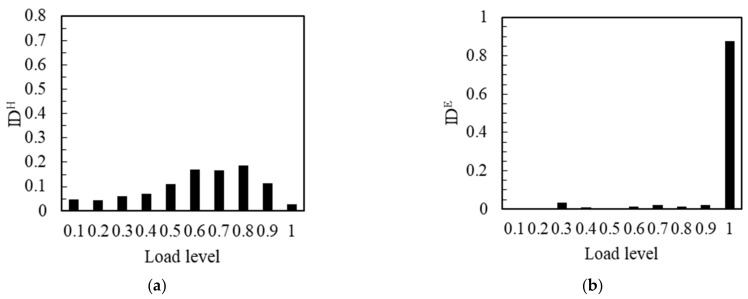
$ID^{H}$ and $ID^{E}$ of the No.6 notched beam: (**a**) $ID^{H}$; (**b**)$\;ID^{E}$.

The $ID^{H}$ corresponding to the flexural strength of the notched beam specimen with the low value of 11.39 MPa is shown in Figure 9a. It can be observed that there was an obvious $ID^{H}$ value from the beginning of loading. The trend of gradual rise was maintained until the 0.8 load level. It revealed that the relative hit number was obviously generated and rose from the beginning of loading to 0.8 load level. When the strength of the notched beam was at a low value, the effective resistance of the notched beam to the load decreased significantly, which led to a significant number of impacts at the beginning of the load, and the impact number continued to increase until the 0.8 load level. When the load reached the level of 0.8, the value of $ID^{H}$ dropped significantly and fell to the extremely low values until 1.0 load level, which meant that the impact number decreased rapidly until it reached the maximum load; the development process of a large number of cracks was more advanced at this stage. As shown in Figure 9b, at the 0.1~0.2 load level, the $ID^{E}$ value was almost unobserved, the energy value was very small at this stage. It was noteworthy that the $ID^{E}$ obviously generated and kept a small value from earlier 0.3 load to 0.9 load level, the energy generated and maintained at a lower level at the 0.3~0.9 load level. There were more cracks developed in the early stage of loading with the notched beam that at a lower strength. Although the energy value was relatively low, it cannot be ignored. When the load exceeded 0.9 load level, the $ID^{E}$ suddenly increased until the maximum load and the energy rose sharply at this stage. A limited number of cracks penetrated and released most of the energy at the last loading level.

#### 3.2.3. Fracture Characteristics Based on *b*-Value

**Figure 10:**
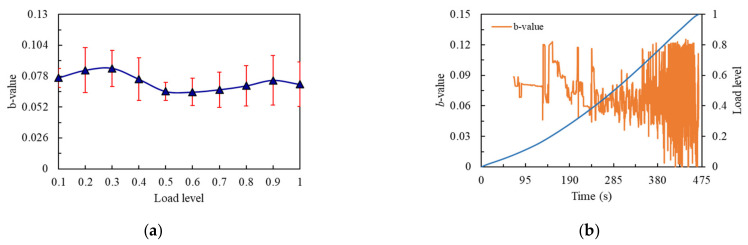
*b*-value curves of the No.14 notched beam: (**a**) *b*-value changes curve with load level; (**b**) *b*-value changes curve with time.

The representative specimens were selected according to the change rule of flexural strengths. As shown in Figure 10a, when the flexural strength of the notched beam specimen was the high value of 15.42 MPa, *b*-value showed a slight upward trend from 0.1 to 0.3 load level, whereby a small amount of micro cracks formed inside the beam at this stage. When load reached 0.3 level, *b*-value showed a downward trend until 0.5 load level. It can be observed that *b*-value kept relatively flat from 0.5 to 0.6 load level. It can be explained that a small number of micro cracks expanded to a certain extent, but the dense internal structure resisted the pressure as early as possible, making the *b*-value enter the transition stage. After 0.6 load level, *b*-value started to show an upward trend until 0.9 load level. When the load exceeded 0.9 level, *b*-value decreased slightly, then the maximum load was reached and the specimen was damaged. From the 0.6 load level, the micro cracks continued to develop and generated large cracks at the last load level, which was also the manifestation of brittleness. From Figure 10b, it can be seen that the overall *b*-value showed a trend of rising first and then declining, and the *b*-value entered a rapid fluctuation around the 0.4~0.5 load level. This coincided with the time when the slope of the cumulative amplitude curve began to increase.

**Figure 11:**
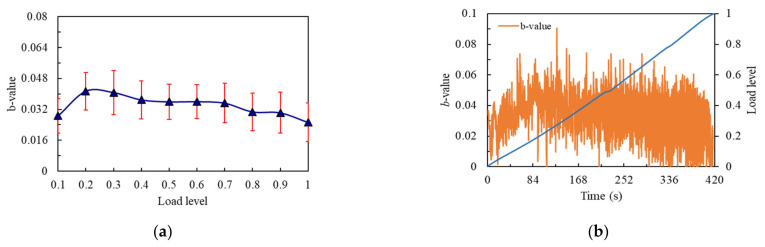
*b*-value curves of the No.11 notched beam: (**a**) *b*-value changes curve with load level; (**b**) *b*-value changes curve with time.

The *b*-value is shown in Figure 11a, whereby the flexural strength of the notched beam specimen was the middle value of 12.83 MPa. The change trend of *b*-value could be roughly divided into two stages. The first stage was the load from 0.1 to 0.2 level. At this time, *b*-value started to rise rapidly in the 0.1 load level until load reached 0.2 level, where micro cracks were produced in the notched beam, the trend only continued at one load level due to the decrease of flexural strength, and then the curve entered the second stage. *b*-value started to decline and maintained a wave decline trend to the last load level. The generation of large cracks was a continuous process, and there was no tendency for micro cracks to regain the upper hand at this stage, although the curve changed in volatility. This was related to the fact that the internal part of the structure was not dense enough or the flexural strength was not high enough, leading to the complex crack development. From Figure 11b, it can be seen that the *b*-value showed a trend of rising first and then falling, and the *b*-value presented a state of rapid fluctuation in the whole process.

**Figure 12:**
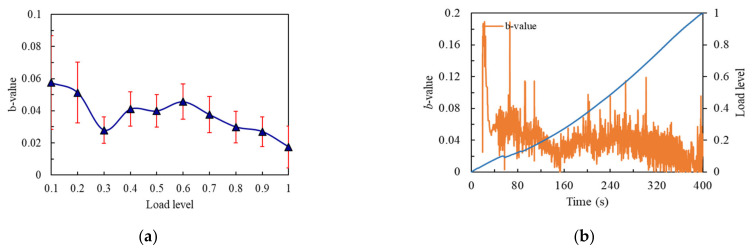
*b*-value curves of the No.6 notched beam: (**a**) *b*-value changes curve with load level; (**b**) *b*-value changes curve with time.

The *b*-value is shown in Figure 12a, whereby the flexural strength of the notched beam specimen was the low value of 11.39 MPa. The change trend of *b*-value could be divided into three stages. The first stage was the load from 0.1 to 0.3 level. The *b*-value declined rapidly at 0.1 load level until 0.3 load level, where it can be found that the large cracks in the internal structure occurred at an earlier load level, which corresponded to the larger value of its energy curve at an earlier time. Then the curve entered the second stage. The *b*-value kept a fluctuating rising trend from 0.3 to 0.6 load level. When the load exceeded 0.6 level, the curve entered the third stage and *b*-value dropped rapidly from 0.6 to the final load level. Although the strength of the notched beam made it show a certain ability to resist the continuous development of large cracks, it entered the rapid development stage of large cracks early at the 0.6 load level because of its lower flexural strength. As can be seen from Figure 12b, the *b*-value curve generally showed a trend of first decreasing and then rising, and then rapidly declining, and the curve maintained strong volatility in the whole process.

## 4. Conclusions

In this paper, the effects of three material factors (water-binder ratio, length of BF, replacement ratio of GS) on the working performance and flexural strength of the UHPC notched beam were studied. The fracture characteristics of UHPC notched beams with different strengths were characterized and compared by using a variety of AE parameters. The following conclusions can be drawn:

(1) The water-binder ratio and the replacement ratio of GS had positive correlations with the working performance of UHPC, while the length of BF had a negative correlation with the slump. A linear slump model can be constructed with correlation coefficients 14.13, −12.75 and 13.63 corresponding to water-binder ratio, length of BF and replacement ratio of GS, respectively.

(2) The water-binder ratio and length of BF had negative correlations with the flexural strength, and the effect of GS replacement on the flexural strength of UHPC notched beam was not obvious. A second-order flexural strength model can be constructed with correlation coefficients −0.9 and −0.66 corresponding to water-binder ratio and length of BF, respectively. The influence degree of the three factors on the flexural strength is: water-binder ratio > length of BF > replacement ratio of GS.

(3) The fracture process of UHPC notched beams with different strengths can be well characterized by AE parameters. When the flexural strength of the notched beam decreased, the amplitude value increased at the early load level and the rapid rise moment of the amplitude advanced. Most of the energy values reflected in the final load level, but the relatively more and larger observable energy values were collected in earlier load levels when the flexural strength decreased.

(4) In the fracture process of the UHPC notched beam, the impact number was mainly reflected in the stage of middle and post load levels, and the impact number rose continuously. When the strength decreased, the higher impact number appeared in the earlier load level, which also had a continuous upward trend. However, at the load levels after the maximum number of impact, the impact number had a downward trend, and the downward time was longer when the flexural strength was lower.

(5) The change rule of *b*-value was a good explanation for the cracks development of UHPC notched beams with different strengths. The *b*-value curve of notched beams with higher strength increased first and then decreased, and it showed an obvious process of resistance to pressure. Whereas the *b*-value curve of notched beams with low strength decreased first and then increased, then continued to decline, and the decrease of *b*-value was more severe.

## Figures and Tables

**Table 1:** Concrete mix design (kg/m^3^).

Group Number	Materials							
	Cement	SF	QS	GS	QP	BF	Water	HRWRA
1	850	195.5	467.5	467.5	331.5	148.75 (13 mm)	161.5	42.5
2	850	195.5	935	0	331.5	148.75 (20 mm)	161.5	42.5
3	850	195.5	467.5	467.5	331.5	148.75 (13 mm)	161.5	42.5
4	850	195.5	0	0	331.5	148.75 (13 mm)	178.5	42.5
5	850	195.5	935	0	331.5	148.75 (13 mm)	178.5	42.5
6	850	195.5	467.5	467.5	331.5	148.75 (20 mm)	178.5	42.5
7	850	195.5	467.5	467.5	331.5	148.75 (20 mm)	144.5	42.5
8	850	195.5	935	0	331.5	148.75 (6 mm)	161.5	42.5
9	850	195.5	467.5	467.5	331.5	148.75 (6 mm)	178.5	42.5
10	850	195.5	935	0	331.5	148.75 (13 mm)	144.5	42.5
11	850	195.5	467.5	467.5	331.5	148.75 (13 mm)	161.5	42.5
12	850	195.5	467.5	467.5	331.5	148.75 (13 mm)	161.5	42.5
13	850	195.5	467.5	467.5	331.5	148.75 (13 mm)	161.5	42.5
14	850	195.5	467.5	467.5	331.5	148.75 (6 mm)	144.5	42.5
15	850	195.5	0	935	331.5	148.75 (6 mm)	161.5	42.5
16	850	195.5	0	935	331.5	148.75 (20 mm)	161.5	42.5
17	850	195.5	0	935	331.5	148.75 (13 mm)	144.5	42.5

**Table 2:** Actual and coded values of the independent variables.

Coded Value	Actual Value		
	*X*_1_	*X*_2_ (mm)	*X*_3_ (%)
−1	0.17	6	0
0	0.19	13	50
1	0.21	20	100

**Table 3:** Experimental results.

Group Number	Preparation Parameters			Responses	
	*X*_1_	*X*_2_ (mm)	*X*_3_ (%)	*Y*_1_ (mm)	*Y*_2_ (MPa)
1	0.19	13	50	71	12.72
2	0.19	20	0	54	11.50
3	0.19	13	50	77	11.43
4	0.21	13	100	94	13.63
5	0.21	13	0	68	13.55
6	0.21	20	50	92	11.39
7	0.17	20	50	58	14.76
8	0.19	6	0	94	13.09
9	0.21	6	50	112	13.28
10	0.17	13	0	44	14.92
11	0.19	13	50	85	12.83
12	0.19	13	50	71	11.75
13	0.19	13	50	74	13.06
14	0.17	6	50	71	15.42
15	0.19	6	100	112	13.30
16	0.19	20	100	83	12.19
17	0.17	13	100	80	13.96

**Table 4:** ANOVA results for models.

Sources	*R* ^2^	Adj. *R*^2^	Adeq. Precision	*F* Value	*p*-Value	Significant
*Y*_1_	0.7992	0.7529	12.434	17.25	<0.0001	Yes
*Y*_2_	0.8377	0.6289	5.895	4.01	<0.05	Yes

**Table 5:** ANOVA results for independent variables.

Sources	*Y*_1_		*Y*_2_	
	*F*	*p*	*F*	*p*
*X*_1_	18.85	0.0008	11.23	0.0122
*X*_2_	15.36	0.0018	5.95	0.0448
*X*_3_	17.54	0.0011	0.000084	0.9929
*X*_12_	-	-	0.65	0.4460
*X*_13_	-	-	0.47	0.5136
*X*_23_	-	-	0.10	0.7581
*X*_11_	-	-	16.50	0.0048
*X*_22_	-	-	0.00041	0.9843
*X*_33_	-	-	0.72	0.4254

## Data Availability

The data presented in this study are available on request from the corresponding author. The data are not publicly available due to a complicated structure that requires additional explanations.
